# Hospital Expenditure.—The Commissariat

**Published:** 1896-08-15

**Authors:** 


### THE INSTITUTIONAL WORKSHOP.

### HOSPITAL EXPENDITURE.—THE COMMISSARIAT.

### XXIII.—THE MILK SUPPLY.

The importance of a good system in every hospital regulating the supply and consumption of milk can hardly be over-rated. In the most serious surgical and medical cases, milk is commonly the only food permitted to the patient, and the comfort and well-being of all the inmates, not excepting the staff, are greatly affected by any deficiency in the quantity and quality supplied. So essential an article of diet, indeed, is it considered, that in most hospitals the supply is liberal even to lavishness, and in no direction is it more difficult to check waste.

The price of milk in London hospitals varies between 7d. and 11d. the imperial gallon, the prices actually paid at the leading hospitals being 7d; 8½d., 8¾d.; 9d. (three hospitals); 9¼d. (two hospitals); 9¾d., and 11d. The first-named price will strike everyone as unfairly inadequate, and likely to lead the way to adulteration. The last-named is unnecessarily high. Here, again, as in the case of the meat supply, a little knowledge would tend to an equalisation of prices. At Middlesex Hospital, where the sensible plan is adopted of separate contracts for summer and winter, the summer price is 7¼d., and the winter one 9¾d.

Provincial prices rule much the same, without, however, touching the minimum quoted above. The lowest is 8d. per gallon at Oxford; then come the Birmingham General, Bristol General, and Sheffield Infirmary at 8½d.; Liverpool Infirmary and Birmingham Queen's at 9d.; Hull, 9½d.; Leeds, 9⅞d.; Brighton (Sussex County), 10d.; Newcastle-on-Tyne, 11d.

In Scotch hospitals the prices range from 8d. at Kilmarnock and Ayr to 10½d. at Edinburgh. Skim milk is a good deal used, and is procured at 3½d., 4¾d., and 6d. per gallon, the latter being the Glasgow price. In Belfast milk costs 9⅜d. per gallon, and at Dublin 9½d.

It will be seen that the prices are not more favourable in provincial towns than in London; but again we are face to face with an enormous disproportion in the amount of this article consumed per head in different institutions, the London hospitals being for the most part conspicuous by a very heavy rate of consumption.

It is very difficult to form a just estimate of the requirements of the patient in this particular, so much depends on the character of the cases presented for treatment, and also on the average length of stay of each patient. Milk diet is commonly prescribed for the first few days of treatment in all serious cases, and if, owing to pressure of space, the patients are forced to move out before becoming fully convalescent, the proportion of those on milk diet, and hence the average consumption of milk per head, is always high. This point is forcibly illustrated by the Birmingham General Hospital, where the admirable secretarial work is well calculated to throw light on vexed questions of hospital finance. The average length of each patient's stay in the Birmingham General, in 1894, was 22 days against 25 or 30 days in many larger institutions. In consequence of this the proportion of those on milk diet is unusually high, viz., 40 per cent., as compared with 8 or 9 per cent. at the Glasgow Western Infirmary, 10 per cent. at Leeds, and 35 per cent. at Bradford. The cost of feeding the patients is therefore unusually low, milk being the least expensive of foods, and the average quantity of milk consumed by each patient is slightly over 1 quart a day; to be quite accurate each patient consumes on an average 2¼ pints a day.

At Guy's the amount of milk found to be consumed by the patient is rather less than a quart a day, viz., 338 quarts a year. Taking all the patients together, and allowing for milk puddings, milk as a beverage, and milk with tea or coffee, it would seem that a quart a day per head is a very liberal reckoning. Convalescent patients, of course, get a great deal less than this, especially where beer or porter is allowed, but then those on low diet may get rather more. It will be seen that a far lower estimate is in many hospitals found to suffice, but it must be remembered that if less milk is used other things, such as beef-tea or beer, must be given. On the whole, then, it may be decided that something less than a quart a day under economical management or with variations of diet, should prove ample for each patient's requirements. More than a quart a day should not be used unless very good reason can be shown for the excess.

The amount required per head for the staff depends solely on the point whether milk is used extensively as a beverage, in place of mineral waters, beer, &c. At Guy's Hospital the household consumption is just under 2 quarts a week per head. At the London Hospital Nursing Home, comprising only nurses and servants, the amount used per head is much the same, viz., 2½ quarts weekly. At the Birmingham General Hospital the amount per head used by the staff is slightly over 3 quarts weekly. It would seem that ¾ pint a day, or from 2½ to 3 quarts a week, should prove ample to meet ordinary requirements, reckoning ¼ pint for tea or coffee and ½ pint in the form of pudding. If milk is used as a beverage for lunch and supper as much as an extra pint a day must be allowed for, but as no household consists entirely of milk drinkers (who are generally in the minority), it is clear that about 5 quarts a head weekly is the outside limit of consumption for the household, even under very liberal management.

Taking, then, 1 quart a day as the standard for the patient's needs, it remains to see how the leading hospitals stand in this respect. It may be noted that the amounts used have been worked out very carefully according to the ratio of prices paid at each hospital; at 9d. a gallon each £1 represents 105 quarts, and so on.

Now, in some hospitals the cost of one quart a day for each patient either absorbs or exceeds the total amount of the milk bill for the year. In these cases it is perfectly clear that a great deal less than this amount is used, since the staff, as well as the patients, have to be reckoned for. At St. George's a quart a day for each patient would leave only about 10 quarts a year each for the staff, and at the Great Northern Central the same estimate would leave them only about 8 quarts a year a head. At University College Hospital, again, the patients, at the same rate, would cost over £50 more than is actually expended on patients and household together. But these are exceptions, at any rate, among London hospitals.

At Charing Cross Hospital the estimate of a quart a day for each patient allows for 2½ quarts a week for each member of the staff, the identical amount used at Guy's, and, as we have seen, the normal quantity for the household supply, where milk is not used as a beverage. At the Metropolitan the surplus for the staff is four quarts, and at the Seamen's nearly 5 quarts weekly, which is not unreasonable, if milk is allowed in lieu of beer or mineral waters.

At Middlesex and the Royal Free the amount rises to 7 quarts a week for each official. It must be remembered, in the case of Middlesex, that the cancer wards make unusual demands on every article of common consumption, so that very probably the patients' requirements rise considerably above a quart a day. At the Royal Free milk is used very freely as a beverage, both by doctors and nurses.

We now come to several hospitals where the quantity used is in excess of all reasonable requirements.

It is hardly possible that over a quart of milk a head daily can be consumed among persons in good health on ordinary diet, and, as we have shown good reason for the fact that this amount is ample for the patient, a leakage where it is exceeded must be supposed to exist somewhere. So unusual an allowance calls at any rate imperatively for a note of explanation appended to the ordinary table of expenditure.

At the London Hospital, after due allowance has been made for the patients, 8 quarts a week each remains over for the doctors and servants, and this in view of the fact that the weekly milk consumption of the nurses in the same institution is no more than 2½ quarts. At St. Mary's, making the same computation, the staff would rejoice in 11½ quarts weekly; at Westminster their share would be nearly 14 quarts; and at St. Thomas's, where nurses and doctors are boarded under a separate account, the household would receive actually 20 quarts per week. But this is, of course, under the circumstances, a misleading way of stating the facts. The surplus quantity, no doubt, melts away among patients. At St. Thomas's an extra ½ pint per head for each patient daily nearly absorbs the whole, and how easy it is for this extra amount to be habitually used without attracting special attention, every one at all acquainted with hospital routine is only too well aware.

It remains to be seen what quantity of milk is used in some of the leading provincial hospitals. The estimate of a quart a day for each patient is obviously far too high in the following hospitals:—

| | | | |
|---|---|---|---|
| Hull Royal Infirmary ... | where | 6,100 | qts. less are used. |
| Leeds General Infirmary... | ,, | 23,343 | ,, ,, |
| Norwich Infirmary ... | ,, | 6,510 | ,, ,, |
| Newcastle-on-Tyne Infimy. | ,, | 9,224 | ,, ,, |
| Sussex County (Brighton) | ,, | 306 | ,, ,, |

At the Bristol General Infirmary the allowance of a quart per head daily for each patient leaves a little over a pint a week for each member of the household. At Birmingham Queen's and at Sheffield Infirmary the staff would receive 2½ quarts per week each. At Oxford the weekly share of the household works out at 3 quarts each; at Liverpool General Infirmary at 3½ quarts each; and at Cambridge Addenbrooke's at 4 quarts each. This last is the highest for provincial hospitals, and, as has been pointed out before, the closing of several wards at Addenbrooke's in the year under examination (1893) raised every article of consumption.

It has not been possible to estimate the quantity of milk taken in Scotch or Irish hospitals, as skim and butter milk are largely used and included in the milk item.

